# Polymorphisms of genes involved in lipid metabolism and risk of chronic kidney disease in Japanese - cross-sectional data from the J-MICC study

**DOI:** 10.1186/1476-511X-13-162

**Published:** 2014-10-14

**Authors:** Asahi Hishida, Kenji Wakai, Mariko Naito, Shino Suma, Tae Sasakabe, Nobuyuki Hamajima, Satoyo Hosono, Mikako Horita, Tanvir Chowdhury Turin, Sadao Suzuki, Tara Sefanya Kairupan, Haruo Mikami, Keizo Ohnaka, Isao Watanabe, Hirokazu Uemura, Michiaki Kubo, Hideo Tanaka, for the J-MICC Study Group

**Affiliations:** Department of Preventive Medicine, Nagoya University Graduate School of Medicine, Nagoya, 466-8550 Japan; Department of Healthcare Administration, Nagoya University Graduate School of Medicine, Nagoya, 466-8550 Japan; Division of Epidemiology and Prevention, Aichi Cancer Center Research Institute, Nagoya, 464-8681 Japan; Department of Preventive Medicine, Faculty of Medicine, Saga University, Saga, 849-8501 Japan; Department of Health Science, Shiga University of Medical Science, Otsu, 520-2192 Japan; Department of Family Medicine, University of Calgary, Calgary, AB T2N 1N4 Canada; Department of Public Health, Nagoya City University Graduate School of Medical Sciences, Nagoya, 467-8601 Japan; Department of International Island and Community Medicine, Kagoshima University Graduate School of Medical and Dental Sciences, Kagoshima, 890-8544 Japan; Department of Community Medicine, Faculty of Medicine, Sam Ratulangi University, Manado, 95115 Indonesia; Division of Epidemiology, Chiba Cancer Center Research Institute, Chiba, 260-8717 Japan; Department of Geriatric Medicine, Graduate School of Medical Sciences, Kyushu University, Fukuoka, 812-8582 Japan; Department of Social Medicine and Cultural Sciences, Kyoto Prefectural University of Medicine, Kyoto, 602-8566 Japan; Department of Preventive Medicine, Institute of Health Biosciences, University of Tokushima Graduate School, Tokushima, 770-8503 Japan; Core for Genomic Medicine, Center for Integrative Medical Sciences, RIKEN, Yokohama, 230-0045 Japan

**Keywords:** Lipid metabolism, Chronic kidney disease, Single nucleotide polymorphism

## Abstract

**Background:**

Chronic kidney disease (CKD) is known to be one of the causes of cardiovascular disease and end-stage renal disease. Among the several treatable risk factors of CKD, that of dyslipidemia is relatively controversial. To clarify the association of polymorphisms in genes involved in lipid metabolism with the risk of CKD in the Japanese population, we used cross-sectional data from the Japan Multi-Institutional Collaborative Cohort (J-MICC) Study.

**Methods:**

A total of 3,268 men and women, aged 35–69 years, were selected from J-MICC Study participants for inclusion in this study. Twenty-eight candidate single nucleotide polymorphisms (SNPs) were selected in 17 genes associated with the risk of lipid metabolism disorders, and genotyping of the subjects was conducted using the multiplex PCR-based invader assay. The prevalence of CKD was determined for stages 3–5 (defined as estimated glomerular filtration rate <60 ml/min/1.73 m^2^).

**Results:**

Logistic regression analysis revealed that SNPs *APOA5* T − 1131C (rs662799), *APOA5* T1259C (rs2266788), *TOMM40* A/G (rs157580), and *CETP* TaqIB (rs708272) were significantly associated with CKD risk in those individuals genotyped, with age- and sex-adjusted odds ratios (ORs) per minor allele (and 95% confidence intervals (CIs)) of OR 1.22 (95% CI: 1.06–1.39), 1.19 (1.03–1.37), 1.27 (1.12–1.45), and 0.81 (0.71–0.92), respectively. Analysis of the gene–environment interaction revealed that body mass index (BMI) was a significant effect modifier for *APOA5* T − 1131C (rs662799) and a marginally significant effect modifier for *APOA5* T/C (rs2266788), with the interaction between BMI ≥30 and individuals with at least one minor allele of each genotype of OR 10.43 (95% CI: 1.29–84.19) and 3.36 (0.87–13.01), respectively.

**Conclusions:**

Four polymorphisms in *APOA5*, *TOMM40*, and *CETP* were shown to be significantly associated with CKD risk, and a significant interaction between the two *APOA5* SNPs and BMI on CKD risk was also demonstrated. This suggests the future possibility of personalized risk estimation for this life-limiting disease.

**Electronic supplementary material:**

The online version of this article (doi:10.1186/1476-511X-13-162) contains supplementary material, which is available to authorized users.

## Background

Chronic kidney disease (CKD) is emerging as a major public health and financial burden worldwide, and the number of affected patients is increasing in East Asian countries such as Japan, where more than 10 million people currently have CKD stage ≥3. CKD is also known to be a cause of cardiovascular disease (CVD) and end-stage renal disease, so prevention of this potentially life-limiting disease is becoming a pressing issue [1]. CKD has a number of treatable risk factors, such as diabetes mellitus, hypertension, glomerular nephritis, and dyslipidemia [2]. Of these, the effect of dyslipidemia on human CKD risk is relatively controversial, although substantial evidence from animal models is supportive of this association [3, 4], suggesting that dyslipidemia plays an important role in the development and progression of CKD. Additionally, data from 4,483 healthy men participating in the Physician’s Health Study showed that elevated total cholesterol, high non-high-density lipoprotein (HDL) cholesterol, a high ratio of total cholesterol to HDL cholesterol, and low HDL cholesterol in particular were significantly associated with an increased risk of developing renal dysfunction with an initial creatinine level <1.5 mg/dl [5]. Some other reports also suggested that blood lipids modify the decline in renal function as well as hypertension [6, 7].

In 2005, we launched the Japan Multi-Institutional Collaborative Cohort (J-MICC) Study, a large genome cohort study to confirm and detect gene–environment interactions in lifestyle-related diseases, particularly cancer [8, 9]. Considering the potentially important roles of lipid metabolisms in the etiology of CKD, we hypothesized that genetic polymorphisms modulating lipid metabolizing pathways would also affect CKD risk in humans. Accordingly, to clarify the association of polymorphisms in genes involved in lipid metabolism with CKD risk, we examined this in Japanese subjects using the cross-sectional data of the J-MICC Study.

## Results

### Subject characteristics and allele frequencies of genes involved in lipid metabolism

Subject characteristics are summarized by CKD status in Table 1. The mean age ± standard deviation was 56.6 ± 8.6 years, and 48.6% of all subjects were men. Subjects with CKD accounted for 17.3% (564/3,268) of the entire study population. Estimated glomerular filtration rate (eGFR), age, systolic blood pressure, total cholesterol, uric acid, use of anti-hypertensive or lipid-lowering drugs, history of CVD or cerebrovascular disease, and current smokers were all significantly different between subjects with and without CKD.

**Table 1:** **Comparison of characteristics between subjects with and without CKD (n = 3,268)**

	CKD (+)	CKD (-)	***P***
	(n = 564)	(n = 2,704)	
CKD stage 3 (eGFR < 60 ml/min/1.73m^2^)	561 (99.5%)	-	-
Stage 4 (eGFR < 30)	0 (0%)	-	
Stage 5 (eGFR < 15)	3 (0.5%)	-	
eGFR (ml/min/1.73m^2^)	53.7 ± 6.0	78.3 ± 12.5	< 0.001
Age (years)	60.4 ± 7.2	55.9 ± 8.7	< 0.001
Male	260 (46.1%)	1,327 (49.1%)	0.198
Body mass index	23.5 ± 3.1	23.4 ± 3.3	0.413
Systolic blood pressure (mm Hg)	130.1 ± 19.8	128.1 ± 19.4	0.029
Diastolic blood pressure (mm Hg)	78.8 ± 12.4	78.6 ± 11.9	0.746
Use of anti-hypertensive drugs	149 (26.4%)	486 (18.0%)	< 0.001
Fasting plasma glucose (mg/dL)	98.8 ± 22.1	100.0 ± 20.8	0.291
HbA1c (%)	5.22 ± 0.70	5.22 ± 0.66	0.942
Use of glucose-lowering drugs	31 (5.5%)	110 (4.1%)	0.129
Total cholesterol (mg/dL)	218.2 ± 34.0	211.1 ± 33.9	< 0.001
HDL cholesterol (mg/dL)	62.0 ± 15.9	63.3 ± 16.3	0.071
Triglyceride (mg/dL)*	106 (76.5-151)	104 (74-154)	0.992
Use of lipid-lowering drugs	70 (12.4%)	225 (8.3%)	0.002
Uric acid (mg/dL)	5.53 ± 1.47	5.10 ± 1.33	< 0.001
History of cardiovascular diseases	34 (6.0%)	80 (3.0%)	<0.001
History of cerebrovascular diseases	29 (5.1%)	52 (1.9%)	< 0.001
Current smokers	69 (12.2%)	484 (17.9%)	0.001
Current drinkers	295 (52.3%)	1,510 (55.8%)	0.138

Values are expressed as means ± standard deviation, n (%), or median (interquartile range). CKD: chronic kidney disease. CKD was defined as estimated glomerular filtration rate <60 ml/min/1.73 m^2^.

*Non-fasting.

The genotype frequencies included in the analyses were in accordance with Hardy–Weinberg equilibrium (HWE), except for the following: apolipoprotein A5 gene (*APOA5*) G553T (Cys185Gly, rs2075291) (*T* allele = 0.066, χ^2^ = 4.791, *P* = 0.029), apolipoprotein E gene (*APOE*) T471C (Cys112Arg, rs429358) (*C* allele = 0.099, χ^2^ = 6.833, *P* = 0.009), *APOE* T − 219G (rs405509) (*G* allele = 0.307, χ^2^ = 8.666, *P* = 0.003), and the translocase of outer mitochondrial membrane 40 homolog gene (*TOMM40*) A/G (rs157580) (*G* allele = 0.473, χ^2^ = 6.626, *P* = 0.010). The genotype call rate was more than 99.6% for all individuals with serum creatinine (SCr) data (*n* = 3,326).

### Polymorphisms involved in lipid metabolism and risk of CKD

The potential confounders tested did not fulfill the significance criteria of change in estimate (CIE) >0.1 (10%), so we adopted the odds ratios (ORs) adjusted only for age and sex. Logistic regression analysis revealed that *APOA5* T − 1131C (rs662799), *APOA5* T1259C (rs2266788), *TOMM40* A/G (rs157580), and cholesterol ester transfer protein gene (*CETP*) TaqIB (rs708272) were significantly associated with the risk of CKD, with age- and sex-adjusted ORs (aORs) and 95% confidence intervals (95% CIs) of aOR 1.22 (95% CI: 1.06–1.39), 1.19 (1.03–1.37), 1.27 (1.12–1.45), and 0.81 (0.71–0.92), respectively (Table 2). Because the two *APOA5* SNPs (rs662799 and rs2266788) are reported to be closely linked, we also conducted haplotype analysis for these loci. This confirmed that the two SNPs were tightly linked, with linkage disequilibrium (LD) coefficients *D’* = 0.99, *r*^*2*^ = 0.72, while the *C-C* haplotype was found to be significantly associated with an increased risk of CKD (aOR 1.18 (95% CI: 1.02–1.36)) (Additional file 1: Table S1). We also conducted the analysis of serum lipid levels according to these genotypes found to be significant, as it may provide important information about the possible underlying mechanisms for the associations found (Additional file 2: Table S2).

**Table 2:** **Polymorphisms in lipid metabolizing genes and risk of CKD**

Polymorphism	Genotype	CKD (+)	CKD (-)	Per allele	***P***
		(n = 564)	(n = 2,704)	aOR (95% CI)*	
*APOA1* Ala61Thr (G219A)	*G/G*	506 (89.7%)	2,423 (89.6%)		
(rs12718465)	*A/G*	56 (9.9%)	268 (9.9%)	0.98 (0.74-1.31)	0.901
	*A/A*	2 (0.4%)	13 (0.5%)		
*APOA5* G553T (Cys185Gly)	*G/G*	483 (85.6%)	2,375 (87.8%)		
(rs2075291)	*G/T*	79 (14.0%)	309 (11.4%)	1.12 (0.87-1.43)	0.378
	*T/T*	2 (0.4%)	20 (0.7%)		
*APOA5* T-1131C	*T/T*	221 (39.2%)	1,197 (44.3%)		
(rs662799)	*C/T*	258 (45.7%)	1,183 (43.8%)	1.22 (1.06-1.39)	*0.004*
	*C/C*	85 (15.1%)	324 (12.0%)		
*APOA5* C/A	*C/C*	210 (37.2%)	1,028 (38.0%)		
(rs6589567)	*C/A*	264 (46.8%)	1,241 (45.9%)	1.03 (0.90-1.17)	0.684
	*A/A*	90 (16.0%)	435 (16.1%)		
*APOA5* T1259C	*T/T*	282 (50.0%)	1,437 (53.1%)		
(rs2266788)	*T/C*	224 (39.7%)	1,059 (39.2%)	1.19 (1.03-1.37)	*0.019*
	*C/C*	58 (10.3%)	208 (7.7%)		
*APOB* A/G	*A/A*	308 (54.6%)	1,421 (52.6%)		
(rs673548)	*A/G*	210 (37.2%)	1,056 (39.1%)	0.95 (0.82-1.10)	0.490
	*G/G*	46 (8.2%)	227 (8.4%)		
*APOE* Arg158Cys (C609T)	*C/C*	523 (92.7%)	2,472 (91.4%)		
(rs7412)	*C/T*	40 (7.1%)	226 (8.4%)	0.82 (0.58-1.15)	0.255
	*T/T*	1 (0.2%)	6 (0.2%)		
*APOE* Cys112Arg (T471C)	*T/T*	469 (83.2%)	2,200 (81.4%)		
(rs429358)	*T/C*	88 (15.6%)	466 (17.2%)	0.91 (0.73-1.13)	0.388
	*C/C*	7 (1.2%)	38 (1.4%)		
*APOE* T-219G	*T/T*	290 (51.4%)	1,317 (48.7%)		
(rs405509)	*T/G*	228 (40.4%)	1,090 (40.3%)	0.90 (0.78-1.03)	0.124
	*G/G*	46 (8.2%)	297 (11.0%)		
*APOE cluster* A/G	*A/A*	452 (80.1%)	2,155 (79.7%)		
(rs4420638)	*A/G*	104 (18.4%)	509 (18.8%)	0.97 (0.79-1.20)	0.798
	*G/G*	8 (1.4%)	40 (1.5%)		
*TOMM40* A/G	*A/A*	118 (20.9%)	825 (30.5%)		
(rs157580)	*A/G*	299 (53.0%)	1,257 (46.5%)	1.27 (1.12-1.45)	*<0.001*
	*G/G*	147 (26.1%)	622 (23.0%)		
*HMGCR* G/A	*G/G*	163 (28.9%)	736 (27.2%)		
(rs3846662)	*G/A*	278 (49.3%)	1,326 (49.0%)	0.91 (0.80-1.04)	0.162
	*A/A*	123 (21.8%)	642 (23.7%)		
*LPL G*/A	*G/G*	366 (64.9%)	1,797 (66.5%)		
(rs331)	*G/A*	177 (31.4%)	816 (30.2%)	1.04 (0.88-1.22)	0.681
	*A/A*	21 (3.7%)	91 (3.4%)		
*LPL* G1791C (Ser474Stop)	*G/G*	423 (75.0%)	2,083 (77.0%)		
(rs328)	*G/C*	131 (23.2%)	579 (21.4%)	1.07 (0.89-1.30)	0.466
	*C/C*	10 (1.8%)	42 (1.6%)		
*NR1H3* G/A	*G/G*	301 (53.4%)	1,512 (55.9%)		
(rs7120118)	*G/A*	224 (39.7%)	1,027 (38.0%)	1.12 (0.97-1.31)	0.125
	*A/A*	39 (6.9%)	165 (6.1%)		
*NR1H3* A/G	*A/A*	299 (53.0%)	1,524 (56.4%)		
(rs2167079)	*A/G*	227 (40.2%)	1,017 (37.6%)	1.14 (0.99-1.33)	0.078
	*G/G*	38 (6.7%)	163 (6.0%)		
*MTNR1B* A/G	*A/A*	250 (44.3%)	1,245 (46.0%)		
(rs1447352)	*A/G*	247 (43.8%)	1,176 (43.5%)	1.06 (0.92-1.22)	0.392
	*G/G*	67 (11.9%)	283 (10.5%)		
*FADS2* C/T	*C/C*	227 (40.2%)	979 (36.2%)		
(rs174570)	*C/T*	250 (44.3%)	1,266 (46.8%)	0.91 (0.80-1.04)	0.167
	*T/T*	87 (15.4%)	459 (17.0%)		
*KCNJ11* A1577G (Ile337Val)	*A/A*	240 (42.6%)	1,068 (39.5%)		
(rs5215)	*A/G*	241 (42.7%)	1,274 (47.1%)	0.95 (0.83-1.09)	0.485
	*G/G*	83 (14.7%)	362 (13.4%)		
*TMEM57* A/G	*A/A*	256 (45.4%)	1,186 (43.9%)		
(rs10903129)	*A/G*	254 (45.0%)	1,234 (45.6%)	0.90 (0.78-1.04)	0.168
	*G/G*	54 (9.6%)	284 (10.5%)		
*DOCK7* A/C	*A/A*	347 (61.5%)	1,542 (57.0%)		
(rs1167998)	*A/C*	188 (33.3%)	996 (36.8%)	0.86 (0.74-1.01)	0.061
	*C/C*	29 (5.1%)	166 (6.1%)		
*CELSR2* C/T	*C/C*	516 (91.5%)	2,413 (89.2%)		
(rs4970834)	*C/T*	46 (8.2%)	280 (10.4%)	0.79 (0.58-1.07)	0.133
	*T/T*	2 (0.4%)	11 (0.4%)		
*LIPC* Val95Met (G340A)	*G/G*	342 (60.6%)	1,616 (59.8%)		
(rs6078)	*G/A*	193 (34.2%)	945 (34.9%)	0.96 (0.82-1.13)	0.643
	*A/A*	29 (5.1%)	143 (5.3%)		
*LIPC* T-514C	*T/T*	132 (23.4%)	731 (27.0%)		
(rs1800588)	*T/C*	290 (51.4%)	1,353 (50.0%)	1.10 (0.97-1.26)	0.143
	*C/C*	142 (25.2%)	620 (22.9%)		
*CETP* TaqIB (G > A)	*G/G*	234 (41.5%)	934 (34.5%)		
(rs708272)	*G/A*	251 (44.5%)	1,315 (48.6%)	0.81 (0.71-0.92)	*0.002*
	*A/A*	79 (14.0%)	455 (16.8%)		
*CETP* G/T	*G/G*	372 (66.0%)	1,692 (62.6%)		
(rs3764261)	*G/T*	170 (30.1%)	872 (32.2%)	0.86 (0.73-1.01)	0.068
	*T/T*	22 (3.9%)	140 (5.2%)		
*CETP* Ile405Val (G > A)	*G/G*	147 (26.1%)	753 (27.8%)		
(rs5882)	*G/A*	277 (49.1%)	1,312 (48.5%)	1.06 (0.93-1.20)	0.403
	*A/A*	140 (24.8%)	639 (23.6%)		
*CETP* A-629C	*A/A*	162 (28.7%)	825 (30.5%)		
(rs1800775)	*A/C*	279 (49.5%)	1,366 (50.5%)	1.10 (0.96-1.25)	0.174
	*C/C*	123 (21.8%)	513 (19.0%)		

*aOR: adjusted odds ratio (adjusted for age and sex); 95% CI: 95% confidence interval; CKD: chronic kidney disease.

To detect the lifestyle factors involved in gene–environment interactions with the genes significantly associated with CKD risk, we evaluated the interaction term for effect measure modification using the Breslow–Day (B–D) test of homogeneity with α = 0.05. Body mass index (BMI) was extracted as the only covariate that significantly contributed to the outcome prediction. Gene–environment interactions were then assessed by the logistic model incorporating a multiplicative interaction term, which revealed that BMI was a significant effect modifier for *APOA5* T − 1131C (rs662799) and a marginally significant effect modifier of *APOA5* T1259C (rs2266788). The interaction between high BMI (≥30) and individuals with at least one minor allele of each SNP was OR 10.43 (95% CI: 1.29–84.19) and 3.36 (0.87–13.01), respectively. Stratified analyses of the CKD risk for the *APOA5* T − 1131C (rs662799) SNP by BMI resulted in a strikingly higher OR for individuals with at least one *C* allele of *APOA5* T − 1131C (rs662799) when only those individuals with BMI ≥30 were included (OR 12.39 (95% CI: 1.55–99.09); Table 3). Additionally, we conducted an exhaustive investigation of the gene–environment interactions for all SNPs tested, which revealed several statistically significant interactions in addition to the one described above (Additional file 3: Table S3).

**Table 3:** **Stratified analyses for the CKD risk associated with** ***APOA5*** **polymorphisms by BMI levels**

Proportion of minor heterozygous plus homozygous (among subjects with all genotypes)		CKD (+)		CKD (-)		OR*	95% CI*	***P***_interaction_^#^
		(n = 564)		(n = 2,704)				
Genotype	BMI	N	(%)	N	(%)			
*APOA5* T-1131C								
(rs662799)								
	≥ 30	15/16	(93.8)	54/95	(56.8)	12.39	1.55-99.09	
	< 30	328/548	(59.9)	1,453/2,609	(55.7)	1.21	0.999-1.47	0.028
*APOA5* T1259C								
(rs2266788)								
	≥ 30	13/16	(81.3)	50/95	(52.6)	3.68	0.97-13.93	
	< 30	269/548	(49.1)	1,217/2,609	(46.6)	1.14	0.95-1.38	0.079

*aOR: adjusted odds ratio (adjusted for age and sex); 95% CI: 95% confidence interval.

^#^OR for interaction =10.43 (95% CI: 1.29-84.19) for *APOA5* rs662799, and 3.36 (95% CI: 0.87-13.01) for *APOA5* rs2266788.

## Discussion

The present study examined the associations between polymorphisms in genes involved in lipid metabolism and the risk of CKD in the Japanese population, and identified a total of four SNPs (two in *APOA5* (T − 1131C [rs662799] and T1259C [rs2266788]), *TOMM40* A/G (rs157580), and *CETP* TaqIB (G > A) (rs708272)) that were significantly associated with CKD risk. While the functional implications of some of these polymorphisms are well-known, others have not yet been sufficiently clarified.

The *APOA5* SNP T − 1131C (rs662799) is located in the *APOA5* promoter region and is thought to modulate gene expression, with minor allele carriers found to have high triglyceride levels. It is also reported to be in strong LD with the *APOA5* SNP rs2266788 [10, 11]. There exist a considerable number of reports supporting these effects of *APOA5* SNPs on blood triglyceride levels [10, 12, 13], and given the potentially important roles of blood triglyceride concentrations in the development of human CKD [13, 14], the modulation of blood triglyceride levels due to these *APOA5* SNPs may contribute to the genesis of CKD in humans. The result of our haplotype analysis of *APOA5* SNPs (rs662799 and rs2266788) suggested the potentially substantial role of rs662779. As this rs662779 (T-1131C) SNP in *APOA5* is located in the promoter region of the *APOA5* gene, it is speculated to modulate the expression of APOA5, although further biological investigations will be required to confirm it.

*TOMM40* is located within 15 kb of *APOE*, and this is reflected by the strong LD of *TOMM40* polymorphisms, including SNP rs157580, with the *ϵ4* allele. *TOMM40* polymorphisms are therefore very interesting targets to study in association with human disorders such as Alzheimer’s disease [15]. A recent genome-wide association study revealed that the *TOMM40* SNP rs157580 was significantly associated with low triglyceride levels [16], although another study reported a significant association between the *TOMM40* rs157580 minor (*G*) allele and increased levels of triglycerides in the Chinese population [17]. Given that population-specific effects appear to exist between different ethnicities in East Asian countries for the same polymorphism [18], our present finding may provide valuable information for future genetic investigations and help prevent publication bias [19].

The *CETP* TaqI B polymorphism was previously shown to be associated with an effect on HDL cholesterol concentrations [20], as well as subsequent CVD risk [21], which is thought to result from LD between this SNP and an as yet unknown functional mutation in the regulatory region of *CETP* [22]. The functional roles of this *CETP* SNP in the regulation of human blood cholesterol levels have been well established by a number of previous studies [23, 24]. Taking into considerations the important roles of blood cholesterol levels in the risk of renal dysfunction, this *CETP* SNP is considered to be involved in the CKD development through the modulation of blood cholesterol concentrations.

To date, only a few associations between SNPs in lipid metabolizing genes and CKD risk have been reported, with recent studies reporting a role for apolipoprotein L1 variants in the risk and progression of CKD in African American populations [25, 26], and associations of the apolipoprotein A1 gene (*APOA1*) and *APOA5* with CKD risk. Associations between the four SNPs and CKD risk have not previously been reported, so this study provides novel evidence for the effect of genetic variations in these genes involved in lipid metabolism and CKD risk. Moreover, a previous significant association observed between *APOE* rs405509 and CKD risk was not replicated in the present study. The associations of some, but not all, of the SNPs in our study with CKD followed a similar trend to that previously reported for CVD [27, 28], which might be expected given that CKD is considered to be a form of CVD. The differences could reflect the existence of etiologies specific to each vascular disease, and different gene–environment or gene–gene interactions between races/ethnicities. Nevertheless, the present research appears to confirm the previously reported findings of the possible influence of lipid disorders on the risk of CKD in humans [3, 22].

SNPs shown to be marginally significant in the present study, liver X receptor-alpha gene (*NRIH3*) rs2167079, dedicator of cytokinesis 7 gene (*DOCK7*) rs1167998, and *CETP* rs70827, suggest a possible involvement of these genes in CKD development. NRIH3 inhibits cholesterol absorption, while CETP mediates the exchange of lipids between lipoproteins, resulting in the net transfer of cholesteryl ester from HDL cholesterol to other lipoproteins. Considering the important roles of these genes in lipid metabolism and subsequent CKD onset, the involvement of their functional polymorphisms in CKD risk seems biologically plausible. However, their marginal significance could have been detected as a result of a type I error, which necessitates further investigation with sufficiently larger sample sizes. The remaining SNPs found not to be associated with CKD risk may not play a major role in CKD development, thus discouraging us from their further investigation.

One of the most marked as well as important findings of the present study is the significant interaction between the *APOA5* SNP and BMI on CKD risk, considering its possible future application in the personalized prevention of CKD. BMI can be regarded as a convenient proxy for energy intake and consumption, as demonstrated by the association between dietary fat and obesity [29], which was represented in a study of the interaction between a polymorphism in the *nitric oxide synthase 3 (NOS3)* gene and BMI on the risk of type 2 diabetes [30] and in other studies [18]. *APOA5* polymorphisms have previously been reported to be associated with elevated serum triglyceride levels by several studies [10, 12, 13], and an interaction between the *APOA5* polymorphism and BMI on high serum triglyceride levels was reported in the East Asian population [18]. Taking these findings into consideration, we speculate that the synergistic effect of obesity and dyslipidemia caused by *APOA5* polymorphisms may confer the increased risk of CKD.

Our additional exhaustive investigations of the gene-environment interactions using all polymorphisms tested revealed several statistically significant associations. Although we didn’t take all these interactions into further considerations in the present study, these findings may suggest the way for our future investigations.

The present study has several potential limitations. Serum lipid levels, such as cholesterol and/or triglyceride, could be considered as covariates to be adjusted; however, they can also be regarded as causal intermediates that link the polymorphisms involved in lipid metabolism and CKD risk. Therefore, because adjusting for even a partially causal intermediate phenotype would incorrectly remove a true association and potentially bias the true association [31], we chose not to adjust for these variables in this study. Second, although the genotype frequencies of some of the SNPs investigated significantly deviated from HWE, the actual differences in the number of subjects for all genotypes compared with that expected from the equilibrium were small (up to 2.5%), or the deviation was caused by the relatively small frequency of the minor allele (<10%). Increasing the sample size may have resulted in more robust findings, but it was not easy to do this because of study design constraints. Third, we chose not to adopt the correction of multiple comparisons by Bonferroni procedures because this study was conducted under an exploratory context, and because such adjustments can be regarded as too conservative [32]. Fourth, all CKD cases were diagnosed from SCr data, which potentially differ from the actual GFR based on renal measurements, so could have diluted the effect of each genotype on CKD risk. Finally, albuminuria was not detected in the present study. Further investigations with improved study designs are therefore required.

## Conclusions

The present study found that two *APOA5* polymorphisms (T − 1131C [rs662799] and T1259C [rs2266788]), as well as *TOMM40* A/G (rs157580) and *CETP* TaqIB (G > A) (rs708272) were significantly associated with CKD risk in the Japanese population. A significant interaction between the *APOA5* T − 1131C SNP and BMI on CKD risk was also demonstrated, indicating the future possibility of personalized risk estimation for this life-limiting disease.

## Methods

### Study subjects

Subjects were participants of the J-MICC Study, initially conducted in 10 areas of Japan, in which around 75,000 voluntarily enrolled participants aged 35–69 years provided blood samples, health check-up data, and lifestyle data through a questionnaire after providing their written informed consent [8].

In the present analysis, 4,519 randomly selected participants (about 500 subjects from each of the 10 areas) were analyzed for whom 108 selected polymorphisms had been genotyped [9]. Of these individuals, six were excluded because of ineligibility or withdrawal from the study. SCr had been measured in 3,326 respondents from eight of the 10 areas of Japan. Of these, 58 were excluded because of genotyping failure, and the remaining 3,268 were included in the analyses. Informed consent was obtained from all subjects and the study protocol was approved by the Institutional Review Board (IRB) of Nagoya University Graduate School of Medicine (IRB approval no. 253-6) and the affiliated Medical Universities.

### Evaluation of lifestyle exposure

Lifestyle exposures were evaluated by a self-administered questionnaire that was checked by trained staff. The questionnaire included items on smoking status, alcohol consumption, and medical history. Smoking status was classified as current, former, or never, and the level of exposure was evaluated in pack-years. Former smokers were defined as people who had quit smoking for at least 1 year. Alcohol consumption for each type of beverage was determined by average intake frequency and quantity, then converted into the Japanese sake unit gou (180 ml), which is equivalent to 23 g of ethanol. The participants were categorized into non-habitual drinkers, habitual drinkers who drank less than 1 gou per day, and those who drank at least 1 gou per day; the latter two groups were coded as indicator variables. Intakes of energy and macronutrients were estimated based on responses to a food frequency questionnaire (FFQ), for which its reproducibility and validity to estimate nutrient intakes had been tested and confirmed [33–36]. Correlation coefficients between the FFQ and 3-day food records were 0.49 for energy, 0.61 for % energy from fat, and 0.86 for % energy from carbohydrate in men. The corresponding figures in women were 0.44, 0.48, and 0.66, respectively [35].

### eGFR and definitions of CKD

SCr was measured in all participants using an enzymatic method. The eGFR of each participant was calculated based on SCr, age, and sex using the following Japanese eGFR equation proposed by the Japanese Society of Nephrology [37]: eGFR (ml/min/1.73 m^2^) = 194 × SCr (mg/dl)^–1.094^ × age^–0.287^ (×0.739 if female). The prevalence of CKD was determined for CKD stages 3–5 (defined as eGFR <60 ml/min/1.73 m^2^).

### Selection of SNPs

We selected 28 candidate SNPs in 17 genes based on the notion that they are well characterized and reported to be associated with the risk of lipid metabolism disorders using public databases such as PubMed and Online Mendelian Inheritance in Man. The selected SNPs were as follows: *APOA1* Ala61Thr (G219A) (rs12718465), *APOA5* G553T (Cys185Gly) (rs2075291), *APOA5* T − 1131C (rs662799), *APOA5* C/A (rs6589567), *APOA5* T1259C (rs2266788), apolipoprotein B gene (*APOB*) A/G (rs673548), *APOE* Arg158Cys (C609T) (rs7412), *APOE* Cys112Arg (T471C) (rs429358), *APOE* T − 219G (rs405509), *APOE cluster* A/G (rs4420638), *TOMM40* A/G (rs157580), 3-hydroxy-3-methylglutaryl-CoA reductase gene (*HMGCR*) G/A (rs3846662), lipoprotein lipase gene (*LPL*) G/A (rs331), *LPL* G1791C (Ser474Stop) (rs328), *NR1H3* G/A (rs7120118), *NR1H3* A/G (rs2167079), melatonin receptor 1B gene (*MTNR1B*) A/G (rs1447352), fatty acid desaturase 2 gene (*FADS2*) C/T (rs174570), potassium channel, subfamily J, member 11 gene (*KCNJ11*) A1577G (Ile337Val) (rs5215), macoilin gene (*TMEM57*) A/G (rs10903129), *DOCK7* A/C (rs1167998), cadherin gene (*CELSR2*) C/T (rs4970834), hepatic lipase gene (*LIPC*) Val95Met (G340A) (rs6078), *LIPC* T − 514C (rs1800588), *CETP* TaqIB (G > A) (rs708272), *CETP* G/T (rs3764261), *CETP* Ile405Val (G > A) (rs5882), and *CETP* A − 629C (rs1800775).

### Genotyping

DNA was extracted from buffy coat using a BioRobot® M48 Workstation (QIAGEN, Tokyo, Japan), or from whole blood samples using an automatic nucleic acid isolation system (NA-3000, Kurabo Industries Ltd., Osaka, Japan). Genotyping was performed by the RIKEN institute (Wako, Japan) using the multiplex PCR-based invader assay (Third Wave Technologies, Madison, WI) as described previously [38].

### Statistical analysis

Differences in the distribution of each characteristic variable between individuals with and without CKD were examined by the Student’s *t*-test or χ^2^ test. Accordance with HWE, indicating an absence of discrepancy between genotype and allele frequencies, was determined using the χ^2^ test. Logistic regression analysis was performed to estimate age- and sex-adjusted ORs and 95% CIs for CKD by genotype. All other potential confounding variables, including BMI, systolic blood pressure, diastolic blood pressure, fasting plasma glucose, glycated hemoglobin, total cholesterol, HDL cholesterol, triglycerides, uric acid, past history of cardiovascular or cerebrovascular diseases, use of anti-hypertensive, glucose-lowering, or lipid-lowering drugs, smoking status, and drinking habit were tested [39] to determine if they produced significant CIEs, as described previously [40, 41].

We next evaluated the interaction term for effect measure modification using the B–D test of homogeneity with α = 0.05 [42]. Using the variables extracted by the process above, gene–environment interactions were assessed by the logistic model, which included a multiplicative interaction term as well as variables for genotypes, environment factors, age, and sex. Age adjustments in the analyses were performed with ages regarded as continuous variables. Analyses by genotype based on per allele model were carried out with genotypes for each polymorphism coded as ordinal–categorical variables according to the number of minor alleles. Differences of serum lipid levels by genotype were analyzed with Kruskal-Wallis test. All *P* values were two-sided, and all calculations were performed using Stata® version 10 software (StataCorp, College Station, TX).

## Authors’ information

We all agree that every author is the co-first author of the paper.

## Electronic supplementary material

Additional file 1: Table S1: Estimated haplotype frequencies of *APOA5* SNPs T-1131C (rs662799) and T1259C (rs2266788) and risk of CKD. (DOC 28 KB)

Additional file 2: Table S2: Lipid profiles according to genotypes of *APOA5* and *TOMM40.* (DOC 31 KB)

Additional file 3: Table S3: Exhaustive interaction analyses for the CKD risk between all tested polymorphisms and lifestyle factors. (DOC 66 KB)

## Abbreviations

CKD: Chronic kidney disease
HDL-C: High-density lipoprotein cholesterol
SCr: Serum creatinine
APOA5: Apolipoprotein A5
CETP: Cholesteryl ester transfer protein
LPL: Lipoprotein lipase
TOMM40: Translocase of the outer mitochondrial membrane 40
OR: Odds ratio
CI: Confidence interval.
