# Supplementary material for: Polymorphisms of genes involved in lipid metabolism and risk of chronic kidney disease in Japanese - cross-sectional data from the J-MICC study
Source: Lipids Health Dis. 2014 Oct 14;13:162. doi: 10.1186/1476-511X-13-162 (PMC4210508; doi:10.1186/1476-511X-13-162)
Supplement: Supplementary file 1 — Additional file 1: Table S1: Estimated haplotype frequencies of APOA5 SNPs T-1131C (rs662799) and T1259C (rs2266788) and risk of CKD. (DOC 28 KB) [file 12944_2014_1143_MOESM1_ESM.doc]

**Additional file 1: Table S1 Estimated haplotype frequencies of *APOA5* SNPs T-1131C (rs662799) and T1259C (rs2266788) and risk of CKD**
