# Supplementary material for: Polymorphisms of genes involved in lipid metabolism and risk of chronic kidney disease in Japanese - cross-sectional data from the J-MICC study
Source: Lipids Health Dis. 2014 Oct 14;13:162. doi: 10.1186/1476-511X-13-162 (PMC4210508; doi:10.1186/1476-511X-13-162)
Supplement: Supplementary file 2 — Additional file 2: Table S2: Lipid profiles according to genotypes of APOA5 and TOMM40. (DOC 31 KB) [file 12944_2014_1143_MOESM2_ESM.doc]

**Additional file 2: Table S2 Lipid profiles according to genotypes of *APOA5* and *TOMM40***

*The number of missing data: 1 for triglyceride and 49 for LDL-C.

^#^: *P* values for the Kruskal-Wallis test.
