# Supplementary material for: Polymorphisms of genes involved in lipid metabolism and risk of chronic kidney disease in Japanese - cross-sectional data from the J-MICC study
Source: Lipids Health Dis. 2014 Oct 14;13:162. doi: 10.1186/1476-511X-13-162 (PMC4210508; doi:10.1186/1476-511X-13-162)
Supplement: Supplementary file 3 — Additional file 3: Table S3: Exhaustive interaction analyses for the CKD risk between all tested polymorphisms and lifestyle factors. (DOC 66 KB) [file 12944_2014_1143_MOESM3_ESM.doc]

**Additional file 3: Table S3 Exhaustive interaction analyses for the CKD risk between all tested polymorphisms and lifestyle factors**

*OR: crude odds ratio.

^#^: *P* values for the Breslow-Day test.
